# The Treatment of Proximate Surfaces

**Published:** 1889-03

**Authors:** Safford G. Perry

**Affiliations:** New York


### THE TREATMENT OF PROXIMATE SURFACES.*

(*Concluded.*)

BY SAFFORD G. PERRY, D.D.S., NEW YORK.

If I shall not weary you too much I will now give a few moments to the consideration of some practical points in operating on the approximal surfaces.

The ideal filling on the approximal surface of a bicuspid or molar, is one that shall be large enough so that on close inspection its margins shall be in sight, and thereby safe from capillary attraction on all sides except along the cervical border. But it shall not be large enough to be seen by the non-professional observer, nor shall it go under the gum, nor tend to weaken the great arch that connects the two domes or cusps of the teeth. It shall not be filed or finished down to a flat surface, but in outline it shall follow the contour of the original tooth.

On such a protected surface decay is no longer possible, and it is not too much to say that such a filling renders the tooth safer than if it had never decayed and been filled at all. This filling is one that can very often be made. If the decay is very slight it will not be easy, and if the teeth are of good structure it will not be advisable to get the free margin. If it is extensive, it will not be possible to save the great grinding surface arch.

The manner in which I make this ideal filling is by applying the dam, adjusting a separator—of the two bar pattern generally, as they are more out of the way—and commence to turn the screw, giving a few moments' rest between each effort. I then select the direction from which the filling will be introduced, and shape the outlines of the cavity in accordance with this general plan. If I enter the cavity from the coronal aspect I am very careful not to cut away too much of the grinding surface arch. If from the lingual side, I cut the opening rather freely, in order to reach the cavity and to have the filling reach well out upon the lingual side, where its borders will be swept clean by the friction of the food and the washing action of the fluids of the mouth.

Those cavities are not so easily reached and filled from the lingual side, but by the aid of large mouth mirrors the work is greatly simplified, by standing erect over the patient and working by reflection.

If I decide to enter from the buccal aspect, I cannot cut so

* Read at the Tenth Anniversary Meeting of the Odontological Society of Pennsylvania, Dec. 13, 1888.

freely in entering the cavity, unless it is on the posterior surface, because of the danger of making a filling that would show. But though I feel constrained to make a smaller opening I have the advantage of being able to fill directly and easily into the cavity.

These cavities I prepare with the smallest burs when the engine is used, and with delicate excavators of the spoon variety that are made with very flat and narrow shanks, and with such peculiar curves that every part of the cavity is easily reached. The idea of these flat shanks, which is embodied in both the excavators and pluggers I use, is one that I got from Dr. Darby. As will be easily seen, it gives the greatest amount of strength to an instrument that can be used through the narrowest space. Unless formed by decay, I make the under cuts in all cavities of this class very slight. I make a retaining pit at the distal end of the cavity, and then proceed to fill with the very delicate pluggers here shown. Most of these pluggers swell at the ends in form of a bell. The ends are flat and have serrations as fine as can be made. For many years I have used pluggers with this peculiar swell at the ends for all forms of cohesive and semi-cohesive gold. They are particularly well suited for crystal gold, of which I use a great deal. This form of point runs through many of the pluggers I use for hand pressure, the automatic, the mechanical, the electric, and in heavier forms for the hand mallet. The shanks of some of the smaller ones are so slender that they can be used with the utmost precision through very narrow spaces.

By the use of these delicate cutting and filling instruments, cavities can be prepared and filled from the grinding surface without imparing the strength of the great arch, or from the buccal side without bringing the gold out so that it can be seen except on close examination. In nearly all cases this can be done by the use of the separator without previous wedging. If I can get space enough to allow a strip of thin sand paper to pass between the teeth I can generally fill them so that when the separator is removed and the teeth have returned to their places the edge of the filling can be just seen on all sides except along the cervical border.

If the teeth are very soft and predisposed to decay, even small cavities, when properly prepared, are likely to become large enough to allow these free margins of the fillings. If the teeth are good it is not necessary to get these free margins, although it is well to do so on the lingual and buccal side if too much cutting is not required. Occasionally teeth will be met with where the conditions are all so favorable that it is not necessary to get a free edge on either side. To cut out the decay and to fill them accurately will be sufficient to render them safe indefinitely.

Through many of these narrow spaces I use Watts' crystal gold in very small pieces. It is a wasteful way in which to use gold, for many of the pieces will be dropped or injured before getting them in the cavity through so narrow a space, but this gold stays where it is fixed and is so soft and adjustable, and works so beautifully if used with these bell shaped pluggers, that I often waste time and gold in order to get the fine result that can be had, as I think, in no other way. Undoubtedly strips of soft or cohesive gold are more readily used through these narrow spaces, but the soft gold does not always stay where it is placed in such shallow cavities, and the cohesive gold is too hard and stiff. The crystal gold occupies a medium position between the two, and possesses the best qualities of both. Nearly all my professional life I have used crystal gold more or less, and I have never seen any reason to distrust it. It is not, however, a gold that will give good results unless it is worked with very great care. Every piece must be fixed and condensed before another piece is added. If used in this patient way not much force is required, and an absolutely certain result may be expected. I am satisfied that those who have failed with it have used it in too large pieces and have expected to get on too rapidly with it.

Some of the small cavities in the back teeth I fill with amalgam, and generally with very good results. During the last two years I have filled many of these with copper amalgam, and with excellent results; excepting, of course, discoloration of the fillings. Whether this material will stand on these sheltered surfaces for long periods of time I cannot say. I have noticed a slight waste of the surfaces of some of my copper amalgam fillings, and I could not tell if it was due to mechanical wear or to a disintegration of the surface by galvanic or chemical action. If to the latter, these sheltered surfaces may not prove favorable to them.

The advantages of this ideal filling are that the shapes of the teeth are perfectly kept; the great arch of enamel that binds the two cusps together is not broken, and yet by extending from the lingual to the buccal side, even if not to a free edge on the grinding surface border, the vulnerable part of the tooth is completely protected. It is well known that decay generally begins just above the point of absolute contact,—in fact, on the surface that would be covered by such a filling as this. Still another advantage is that the gum is not disturbed in the least. At the cervical border I do not cut under the gum as much as formerly, but I leave the diminishing enamel, whenever it can possibly be left, for I can never hope to carry the filling under the gum and finish it so as to get quite as good a condition of this easily inflamed tissue as when it rests on its native enamel.

It must be remembered that in packing along the cervical border the force is applied directly against the enamel edge, and if it is always done by gentle hand pressure and with soft forms of gold, the enamel, which grows so thin as it goes under the gum, would not be shattered and destroyed, as it is almost certain to be if the mallet and cohesive gold are used. The delicacy of the small pluggers shown will indicate how gentle this pressure must be, as much force would be certain to break them. I have them made delicate partly to insure this careful use of them along this border.

I want to call particular attention to this filling that I have ventured to call the ideal one. There are some very interesting facts grouped about it, and I cannot resist the impulse to sound the alarm in reference to a method of making these fillings, which, like the practice of making permanent separations, is full of temptations and dangers. I refer to what I have already alluded to and condemned, the habit of cutting boldly down from the grinding surface in order to get at the cavities, and to be able to fill them more rapidly and more easily. As with permanent separations, though in nothing like the same degree, I speak with sad experience of this practice. In that early period before alluded to I used the Varney pluggers for most operations, and as they are nearly all straight instruments, with heavy shanks, it was necessary to open freely into all cavities to be filled with them. It was also my habit then to use more cohesive gold than I use to-day, and of course this necessitated a free, wide opening into all cavities. The result of this practice was, in the first place, a great deal of cutting, and in the second place a great deal of filling; and then a great deal of finishing. And after it was all done, although there was great strength and promise of durability, yet there was a great mass of gold and an artificial condition that was not pleasing to contemplate. The result of that practice leaves many bicuspids filled on both approximal surfaces, the great arches gone, the fissure between the two fillings filled, and the great domes of the cusps standing alone, greatly weakened and ready to split off if a shot from a game bird or a splinter of bone from a chop is caught and wedged between them by the sledge-hammer blows of the lower jaws. The enamel does not coalesce in the fissure, so that the strength lies in what I will call the enamel rim around it. If this rim is cut, as is generally done in filling, even small cavities on the approximal surfaces, much strength is irretrievably lost, and the first step in the downward course of the tooth is taken.

In these days of matrices there are great temptations to cut through this rim, even for the filling of small cavities.

Separators, with all their possibilities for good in careful hands, must also come in for a share of blame here, for as men abandon the habit of preliminary wedging and depend upon the separator, in addition to the slight space that can comfortably be made with the screw, there is the temptation to take a little off from the grinding surface border of the tooth in order to reach the cavity and complete the operation at a single sitting. This is an ever present temptation, and is so potent ,that with firm teeth, I often guard against it by still resorting to slight preliminary wedging with tape, two or three days before operating.

If the cavities are so large that this rim cannot be saved and the ideal filling made, then of course there is no escape from cutting boldly, and in most cases to and including the fissure. There are not many cases where the fissure is reached that it is not best to cut it out to its extremity and fill it in connection with the approximal surface. If left unfilled it may cause a leak that will undermine the whole approximal filling. For some years past it has been my practice to save this coronal arch whenever it was possible, even if it involved more work and more care. And with children it is very common with me to open the permanent teeth with a separator, and cutting out the decay with the delicate instruments before mentioned, fill them with red gutta-percha, expecting to wait until the teeth have become more dense and in better condition to receive gold fillings. It is sometimes surprising to see how many years these little ones will last on these sheltered surfaces, and without danger of wedging and displacing the teeth from the expansion of the filing, as there is so little of it. It is also surprising to note how the teeth improve in the meantime. But if the cavities are large this is often a wretched practice, as the expansion of the gutta-percha displaces the teeth so that if filled again with gold or amalgam there will be a long period of time when the space so made will give all the annoyance of those made by permanent separations. I have seen a few instances with adults where the teeth never returned to their proper places, and being beyond the reach of exaggerated contour fillings, gave no end of permanent trouble. If the approximal cavities are large and, I desire to use, for children or adults, a plastic other than amalgam, to avoid the above danger I use a little gutta-percha—sometimes amalgam—near the gum and fill the rest of the cavity with oxy phosphate. This method of treating approximal surfaces, first undertaken to carry patients over the spring until autumn, or from the holidays until the summer vacation, has so often exceeded my expectations that for many years I have adopted it as one of my means for regular and somewhat permanent treatment for young, soft teeth, and for frail and crumbling teeth of any age. If senile conditions exist I resort to it constantly.

If there are dentists who make permanent separations because they doubt their own ability to make lasting contour fillings of gold, I think they would do better for their patients by adopting this plan; for by it they could easily preserve the shapes of the teeth.

It will sometimes happen, in opening into a cavity from the buccal or lingual aspect in order to save the coronal arch, that a very thin matrix of steel, platinum or German silver can be used to great advantage when adjusted and tied, as shown in a case I will hand around. If the opening into the cavity can be a large one, this matrix is nearly perfect; but if the opening is small the matrix, however thin, takes up room, and is an obstruction rather than an advantage.

It can be sometimes used to very great advantage in filling good sized cavities with soft gold in the molars where it is desirable to save the coronal arch. For amalgam, gutta-percha or oxyphosphate in these places it is indispensable. Also for filling the approximal surfaces of the incisors with any material whatever when the cavities are opened from the under side it is of the very greatest value. The packing of crystal gold, No. 1 or 2, by hand pressure, into a cavity so prepared, standing upright over the patient and using such a reflecting mirror as is here shown, is an operation that gives me more pleasure than any other operation in dentistry. It is the easiest, as it is the most accurate way, in which such a cavity can be filled. The matrix prevents the possibility of the instrument slipping through, and the gold can be packed against the enamel borders in a manner to satisfy the most exacting. The bell-shaped points and nearly right angle curved instruments work to perfection here, as in so many other cavities. The form of the tooth is kept, and when the matrix is removed but little finishing will be required. A thin, flat, highly polished burnisher rubbed on a moist piece of soap, and carried with a firm hand along the edge of the filling, will leave a margin that will make one bless the day when this beautiful form of gold was produced. I could almost honor Dr. Dwinelle as much for the share he had in discovering and producing this form of gold as for the pictures engraved by his own hand illustrating in the old *American Journal of Dental Science* his early contour fillings. In fact, I do not know but that the two are so interwoven that they should be grouped together when placed to his credit. I do not think crystal gold is suited for a hasty, careless operation; but for one who aims to be a fine artist in filling teeth it is, in my judgment, the most perfect form of gold ever produced. But the care necessary in its use will make a good filling of almost any kind of gold; and, after all, it is the result we should care for, and not the individual preference of the operator, or the manner in which the final result is reached.

When the decay on the approximal surfaces of bicuspids and molars has gone so far that the great arch cannot be saved, and the cavity must be opened from the grinding surface, the question of using a matrix is the one first to consider.

From the first introduction of matrices by Dr. Jack until the last few years, I have been rather shy of them; but at the present time I have come to use them in some form or another in most cases where the opening into the cavity is a free one.

The form I use most perhaps is a very thin one of steel, which I described when the separators were brought out. This matrix I use of different widths. If the opening into the cavity is not very free and there is danger that a matrix will shut out the light, and obstruct the cavity, I use only a narrow band along the cervical wall. This serves as a guide in packing the gold and prevents the plugger from slipping over the cervical edge. If the opening into the cavity is large I use a matrix of full width and let it serve as my guide in reproducing the shape of the tooth. These matrices I hold in place with the separators, which clasp them near the gum and hold them near the cervical wall where they most need support. Sometimes, in addition, I secure them at the cervical wall with a simple wedge of wood.

I have also devised a means of holding them in place by the use of a stiff rubber dam clamp, on the jaws of which are soldered lugs or wedges which hold them firmly by means of the spring of the clamps. I have also a form which is made more adjustable by allowing the lugs to rotate. This is in the form of the bail of a pail, and can be turned down out of the way either in front or back of the cavity.

Another form of this holder, which I have used for several years with great satisfaction, is made in the same manner, but is fastened by a screw, which holds it more securely than is done by the clamp or the bail. Perhaps the greatest merit of this device is that it is very quickly applied. It is always ready, almost always fits, and is put on or off in an instant. I have very often used with the greatest satisfaction the first simple matrix devised by Dr. J. A. Woodward. It is rather thick and very strong, and where there is room to apply it, it is one of the most satisfactory that I have ever used. Another matrix, devised by Dr. W. A. Woodward and held by screws impinging on the adjoining tooth I have used countless times with the greatest satisfaction. By loosening the screws gradually the filling can be bulged without unseating the matrix. Of course all these matrices depend upon the adjoining tooth for support. Dr. Guilford's as well as Dr. Brophey's matrices have the merit of depending for support only upon the tooth operated upon, and are of great value. I have devised a matrix which is held also by the tooth being operated on by tying with floss silk, or brass wire, which is threaded through holes at the ends. But Dr. Klapp recently exhibited in New York a somewhat similar one, though he uses a single thread of silk, which is threaded through the two holes and passed several times around the tooth and the matrix and then tied. This holds the matrix closely at the cervical wall, and it is a decided improvement over my method of tying it. Sometime since Dr. Andrews described a method of tying a matrix, but I do not remember that he made holes through it in this way.

I have sometimes used Dr. Klapp's matrix when I thought no other form would have done so well. It is so simple that the wonder is that it had not been thought of before. With these, as with other improvements in our art, complex and laborious means are often first tried only to be laid aside for those more simple and direct. In the use of any matrix, the whole attention must be ap plied to the periphery of the cavity—the ultimate point where the matrix and the edge of the enamel meet. The most serious objec tion to the use of any matrix whatever is the great danger of failing to get a solid condition of the gold here. My fear of failure at this point is so great that after all I fill many cavities without any matrix at all. I can then see and know that I have my edges perfect as I go. In starting a large filling it is the practice of many operators to use soft gold in large masses, prepared either in the form of ropes, strips or cylinders. I doubt somewhat the wisdom of this plan under all circumstances. There is the temptation to use the gold in such large masses that it will not be thoroughly condensed, and there is also danger of the movement of the gold unless it is very firmly and carefully held until the filling is well advanced. If the gold is not solid along the cervical border it will not finish in such a manner as to permanently protect the edges. If the different forms of mallets are used here the force may be sufficient to shatter the enamel border. This I think is a most common occurrence, particularly when that invaluable instrument, the automatic mallet, is injudiciously used here. If one or two small retaining points are made, and crystal gold in small pieces is packed here with rather delicate instruments and hand pressure, a perfect adaptation can be easily made, and when the filling is finally finished, the gold along the cervical border will be solid, and will cut and finish like coin. After a little gold is packed along this delicate border by hand pressure, any one of the mallets may be used to great advantage. The Bonwill mechanical mallet is a marvellous instrument, and, in my judgment, is the most valuable of them all. I never use it in large operations without feeling that its inventor has conferred a lasting benefit upon the profession. For crystal gold it is perfection. I consider it more difficult to get a good adaptation along the side walls than along the cervical border, where the gold is packed directly against the walls. In cases where one must work through a narrow opening, and where the gold must be packed almost by guess, I should prefer the foil to crystal gold, and should try to get the best result I could with strips packed by steady hand pressure and then followed by the automatic mallet.

I do not expect my objection to the use of large masses of soft gold along the cervical border, packed with great mallet force, will meet with favor. But here again I must fall back upon my own experience, and I must say that the old fillings which have lasted so well were almost invariably made with cohesive gold throughout.

Cohesive gold encourages a patient careful habit; while soft gold leads one into a hurried and careless one.

There is not time to give attention to the approximal surfaces of the front teeth. I can only say that almost invariably I fill them from the lingual side. Undoubtedly the Arthur separations can be made on the under surfaces of these teeth to greater advantage than between any other teeth in the mouth; but I do not like to weaken the under plate of enamel, and I generally cut through it only a channel the width of the diameter of the cavity. This lays the cavity open so completely that it can be prepared and filled most easily and most accurately. I never under any circumstances sacrifice any of the enamel on the labial side. Nothing is more painful to me than to see the gold from that side.

In the care of the approximal surfaces of the temporary teeth, I endeavor to follow the general plan here described ; except that with the front teeth I make free spaces up to the gum. I do this with these teeth sometimes in anticipation of decay. I have never filled a temporary tooth with gold since I have been in practice.

The back teeth I fill with copper amalgam, being careful on the approximal surfaces to preserve their shapes, so that the little tots can eat and grow in comfort, unconscious of the possibilities of dentistry.

In operating on the approximal surfaces of any of the teeth, the matter of light and illumination is of the very first importance. I have devised an attachment to the operating chair for holding a condensing lens, which on dark days I find of the greatest value. I also use it for concentrating gas-light at night. The lens is four inches in diameter, and of about twelve inches focus. I have had the good fortune to find at an opticians small concave, reflecting mirrors, two inches in diameter and of about three inch focus, that, in connection with the S. S. White "Jumbo" glass, have proven to be a most valuable acquisition. After becoming accustomed to these, I should not be willing to go back to the ordinary means of illumination.

I am not willing to leave the subject of the approximal surfaces of the bicuspids and molars without calling attention to several important points that I think are often overlooked. In restoring the contour of badly decayed teeth or those that have been badly cut away, I have many times endeavored to save work and compromise by disregarding strict contour and have built out an abutment, that, touching the centre of the adjoining tooth at a single point should hold the teeth in position and protect the gum. Such a form of filling holds the teeth in position, but it does not always protect the gum. Unless the filling is bulged the whole width of the tooth, and rests against the adjoining tooth by a broad surface, the festoons of the gums will be exposed and kept in an irritated and disturbed condition by the impact of food.

Another error is that of building the filling rather scant from the gum to near the grinding surface and then making a rather sudden swell of the filling in order to secure the point of contact. This leaves an ugly space between the tooth above the point of contact which catches food and causes annoyance.

So that at last it comes to be seen that to get the best result we must follow the natural outline of the tooth in each particular case. Each mouth has its own pattern that must be regarded, and accepted as the true guide in shaping the fillings.

All that has been said at such length in this paper could have been put in the single sentence "get free edges, if possible, for your approximal filling, and shape them to the original outline of the tooth." An hour's time would have been saved to you, and you would have been spared the weary task of waiting for the end of a paper that after all can say no more than this.

If the profession is not ready for the acceptance of such a proposition, it will be so in time, for we can no more check its onward growth than we can disarrange the order of the Universe. The marvellous progress of the past will be continued in the future. Nothing of real value will be lost to us. Appliances and conveniences will drop out only as they are superseded by those more valuable. And so we shall go on until the time will be, if it is not yet, when the natural shapes of the teeth will be our guide. We can never exceed this high standard, but we will never be content until it is attained. Those who cannot work up to this standard will give way to those who can, for the inexorable law of the survival of the fittest operates in this as in all other fields of effort.

It does not yet enter into the minds of any of us to conceive the future greatness of our profession. Macauley's New Zealander will never sit upon *its* London Bridge wondering where all its glory and greatness are gone. Built upon the needs of human kind it will endure while those needs last.

